# Repertory of the Symptoms of the Ovaries

**Published:** 1893-10

**Authors:** Cyrus M. Boger

**Affiliations:** Parkersburg, W. Va.


### REPERTORY OF THE SYMPTOMS OF THE OVARIES.

CYRUS M. BOGER, M. D., PARKERSBURG, W. VA.

Abscess of ovaries (compare Suppuration). Crot-h., *Hep.*, *Lach.* (*l*), Merc., PLAT., Psor., Sil.

Aching in ovaries. Lill-t., Medorr. (*l*), Onos., Ovi-gall-p. (*r*).

— — region of ovaries. *Lact-ac.* (*r*), Pic-ac. (*l*), Pod. (*l*), Syph. (*l*).

Acute pain in ovaries. See Sharp.

Alternating pain, between eye and ovary. Sulph.

— — in, and headache. Ovi-gall-p. (*l*).

Anæmia of. FERR., Graph.

Atony of. Alet., *Eupat-purp.*, (Helon.), Puls.

Atrophy of. *Bar-m.*, CON., Helon., IOD., Plb.

Ball, ovary feels like a heavy ball. Carb-an. (*r*).

Bearing down in ovaries. Apis, *Lill-t.* (*l*).

— — — region of ovaries. Apis, (*r*), *Canth.*, Ham., Lac-def., *Lach.* (*l*), Mag-m.

Bearing down pain in left ovary extending up back and down left thigh. Arg-m.

Beating, sensation as if uterus were beating against right ovary. August. (*r*).

Boring in ovaries. Brom. (*l*), *Coloc.*, Lach., Lyc., Sumbul. (*l*), Thuja (*l*), ZINC (*l*).

Bruised sensation in ovaries. Apis (*r*), Arg-m. (*l*), *Therid.*

Bubbling in ovaries. Medorr. (*r*).

Burning in ovaries. Abrot. (*l*), Ananth-m., APIS, ARS. (*r*), *Bufo.*, *Canth.*, (Con.), Eupion (*l*), Goss. (*l*), Lach. (*l*), Lill-t., Lyc., Plat., Sep., *Thu.* (*l*), Ustl., Zinc (*l*).

— — region of ovaries. Ars., *Bell.* (worse *r*), Canth., Carb-an., Coloc. (*r*), *Kali-iod.* (worse *r*), Kali-n. (*r*), Lac-can., Lill-t. (*r*), Medorr. (*l*), Nat-m., Plat., Zinc.

— — paroxysms in ovaries. Plat.

— heat in. Medorr. (*l*).

Burst, sensation as if ovaries would. Graph. (*r*), Med. (*l*).

Cancer of ovaries. Ars., Graph., Kreos., Lapis-alb., Psor.

Coldness, sensation of, in ovaries. Ferr-iod. (*l*).

Colic in ovaries. Apis (*r*), Lach. (*l*), Coloc.

Conjestion of ovaries. Acon., *Alet.*, Apis, *Bell.*, Coni., Ham., Hep., Iod., *Kali-iod.* (*r*), Lac-can. (*r*), Lach., Lill-t., *Magnol.* (*l*), Melil., Naj., Plat., Polygon., Puls., Rhus, Sabin., *Sec-c.* (*r*), Sep., Staph., Sulph., Syphil., Thuj., Zinc.

Constriction in ovaries (compare Grasping). Cact.

Constrictive pain in region of ovaries. Puls. (*r*).

Corkscrew pains. See Boring.

Cramping in region of ovaries. (Compare Colic, Constriction, etc.). Bufo, Coccl., *Coloc.* (*l*), Cub. (*r*).

Cramps in ovaries. Coloc., *Naja.* (*l*).

Crawling in ovaries. Hep.

Cutting in ovaries. Apis, Atrop. (*l*), Bell. (*r*), Canth., Collins (*r*), COLOC., Con., Cub., Graph. (*l*), Ham., Lill-t., Naja, Onos., *Sabad.*, Stram., Syph. (*l*).

— — region of ovaries. *Apis* (*l*), Arg-n. (*r*), Ars., *Arum-tri.* (*l*), Bor., Bry., Canth., *Coccl.*, Collin., Eupat-purp. (*l*), Lyc., Medorr., Nat-m., Phos. (*l*), *Puls.* (*l*), Thuja, Xanth. (*r*).

Cyst of ovaries. Apis, *Bov.*, Canth., *Coloc.*, IOD., *Kali-brom.*, Prun-sp., PLAT., Rhod. (*r*), Rhus-t., Thuja.

Darting in ovaries. Absinth. (*r*), MAG-PHOS. (*r*), Sep. (*r*).

— pain in region of ovaries. Abrotan. (*l*), Bell., Lach. (*l*), Lill-t., Phos., Syph., Thuja.

Debility in ovaries. Chenop-v.

Dislocated, pain in ovaries as if. Apis.

Distention (compare Enlargement, etc.). Aur-mur-nat.

— sensation of in ovaries. Curar., Medorr. (*l*).

Distressing pain. Thuj. (*l*), Ustl.

Dragging in ovaries. BRY. (*r*), Lill-t. (*r*).

— — region of ovaries. Lac-def. (*l*), Plat. (*l*), POD.

Drawing, painful in ovaries. Atrop. (*l*), Bell. (*r*), Coloc. (*l*), Lill-t. (*l*), PLAT., Pod. (*r*).

— in region of ovaries. Apis (*r*), ARS. (*r*), Chin., Goss., Lach. (*r*).

— — — — — toward uterus. Goss.

— downward and forward. Pallad. (*r*).

Dropsy of ovaries. APIS, Arn., *Ars.*, *Aur-mur-nat.*, Bell., Bry., Calc-c., Carb-an., Chin., Coloc., Con., Ferr-iod. (*r*), Graph., *Iod.*, Kali-bro., Kali-c., Kreos., Lach., LILL-T., LYC., Merc., Nat-s., Phos., Plat., Pod., Prun-sp., Rhod., Rhus-t., Sabin., Tereb., Zinc.

Dull pains in ovaries. Amm-bro. (*l*), Brom., Con., Iod., Lill-t. (*l*), Sep.

— — — region of. Apis (*r*), BROM. (*l*), *Ferr-phos.*, Hydrocot., KREOS.

Enlarged sensation of. Arg-m. (*l*), Arg-nit. (*r*), Medorr. (*l*).

Enlargement of. (Compare Swollen, Distention, etc.). APIS (*r*), *Aur-mur-nat.*, BELL. (*r*), CON., *Graph.* (*l*), *Iod.*, Hep., Kali-bro., Lac-can (*l*), Lach. (*r*), *Lill-t.* (*l*), Melil. (*l*), Spong., Ustl. (*l*).

Fluttering in. Brachy. (*r*).

Fullness in. Syphil.

Gnawing in. Coloc. (*l*), Lill-t. (*r*).

Grasping in. Lill-t. (*l*).

Grinding in. Graph. (*r*), Fluor-ac. (*r*).

Griping (compare Colic). Curar. (*r*), Lill-t.

Hardness of (compare Induration). Amyl-n., APIS (*r*), *Brom.* (*l*), Graph. (*l*), Lach. (*l*), *Ustl.* (*l*).

Heat in (compare Burning). *Bufo.*, Medorr. (*l*).

— — region of. Lac-can.

Heaviness of (compare Weight). APIS (*l*), Carb-an. (*r*), Eupat-purp. (*l*), Helon., Kali-c., Lill-t. (*l*), Melil., Onosmod., Sep.

Heavy pains from ovarian region to shoulder-blade with gastralgia (after menses). Borax.

Hernia of. Coccl., Con., Mag-m., Nux-v., Sil., Sulph.

Hydatids of. *Bufo.*, Canth., Merc.

Hypertrophy of (compare Swollen, etc.). *Carb-an.*, Iod., Lactuca.

Induration of (compare Hardness, etc.). Amm-bro. (*l*), APIS, Aur-mur-nat., Ars., *Bar-m.*, Bar-iod., Bell., Brom., Carb-an. (*r*), Con., GRAPH. (*l*), IOD., LACH. (*l*), *Pallad.* (*r*), Plat., *Psor.* (*l*), *Sep.*, Spong.

Inflammation of. Acon., Æscul. (*r*), *Amm-bro.*, Ambr., Ant-cr., Apis (*r*), Arg-m., Arn., Ars., Aur., BELL. (*r*), Brom., Bry., Cact., Canth., Caps. (*l*), *Chin.*, Cimic., Coloc., Con., Crot-hor., Cub., Dulc., Euphorb., Graph. (*l*), Guai., Ham., Igt., Iod. (*r*), Lac-can., *Lach.* (*l*), Lill-t., LYC. (*r*), Mag-phos. *Merc.*, Nit-ac., Nux-v., *Pallad.* (*r*), Plat., Phos-ac., PHOS., Phyt., POD. (*r*), *Puls.*, Rhus-t., Sabad., SABIN., Staph., Syph., Thuja (*l*), Ustl., *Verat-v.*, Znc. (*l*).

Insupportable pain. Alumina (*l*).

Intermittent pains. Ustl. (*l*).

Irritation of. *Amm-bro.*, *Apis*, Bry. (*r*), Carb-ac. (*l*), Cimic., *Gels.*, *Ham.*, Kali-bro., Lill-t., Nux-v., Phyt., Plat., Rhus-t. (*r*), Thuj., USTL., VIBUR.

Itching in region of. Prun-sp.

Jerking in and above. Eupat-purp. (*l*).

Lancinating. See Cutting.

Left ovary. Abrot., Æscul., Alumin., *Amm-br.*, *Apis*, *Arg-m.*, Arg-nit., Artem., Arum-tri., *Atrop.*, BROM., *Caps.*, *Carb-ac.*, Cenchris, Cham., Cimic., COLOC., Cup-ars., Eupat-purp., Eupion, Ferr., Ferr-iod., Ferr-phos., Goss., GRAPH,

Ham., Hydrast., Iod., Kali-bro., Lac-can., Lac-def., *LACH.*, *LILL-T.*, Lyc., Lyssin., Magnol., MEDORR., Melil., Merc., Murex., Naj., Onosmod., Ovi-gal-p., Pic-ac., Phos., PLAT., Pod., Psor., Puls., Sep., Stann., Sumbul., SYPHIL., Therid., THUJ., *USTL.*, *Vesp.*, Wyeth., Xan., *Znc.*, *Zizia.*

Lump in region of. Apis (*l*); (compare Enlargement, Swelling, etc.).

Movement in. Apis.

Needles as of in. Coloc.

Neuralgia of. *Amm-br.* (*l*), Amm-m., Apis (*r*), ATROP., Bell. (*r*), Cham., Cimic. (*l*), Coccl., Collin., COLOC., *Crot-hor.*, Gels., *Ham.*, *Igt.*, KALI-BRO., Kali-phos., Lac-can., LACH (*l*), Lill-t. (*l*), Lyc., *MAG-PHOS.* (*r*), *Naj.* (*l*), Pallad. (*r*), Phyt., Plat., Puls., *Ran-b.*, Sac-lac., Stann., Staph., Tarent., Therid., Urtica., Ustl. (*l*), Xanth. (*l*), Znc. (*l*), Znc-val., Ziz. (*l*).

— in region of. Medorr.

Numbness in region of. Apis (*r*), POD. (*l*).

Obliterated almost. Carbon-sul.

Oppressive pain in. Lill-t. (*r*), Melil.

Pain in (undefined). Acon., Æscul. (*l*), Apis (*r*), *Arg-m.* (*l*), Arg-nit., Ars., Atrop., Aur., BELL. (*r*), Brom. (*l*), Bry. (*r*), Cact., Calc. (*r*), Canth., Cenchris., Cimic., Collin., Coloc. (*l*), Con., Cop., Gels., Graph. (*r*), Guai., *Ham.*, *Helon.*, *Iod.* (*r*), *Kali-bro.* (*l*), Kali-phos., Lac-can., LACH. (*l*), Lill-t. (*l*), Medorr. (*l*), Naja., Onosmod., Ov-gall-p. (*l*), Pallad (*r*), Phos. (*l*), Plat. (*l*), POD. (*r*), Rhod., Rhus-t., Sabad., Sarsap., Sec-c., Sep. (*l*), Staph., Sulph., Syphil. (*r*), Therid. (*l*), Thuj. (*l*), USTL. (*l*), Vesp. (*l*), Vibur., Wyeth (*l*), XAN., Zinc (*l*).

— — in region of. Carb-ac. (*l*), Hell. (*r*), Hydrocot., Lyssin (*l*), Nat-m. (*r*), Vibur., Znc.

— — — — — insupportable. Alumin. (*l*).

— direction of.

Extending across lower part of ABDOMEN from region of. Cimic., Lac-can. (*l*), Lill-t. (*l*).

Extending up to ABDOMEN from left ovary. Ham, Lill-t.

— through ABDOMEN, hips, and back. Con.

— through small of BACK from left ovary. ÆSCUL., Plat. (*l*).

Extending to *Back*. Abrotan., Sulph., Xanth. (*r*),
— to CHEST from ovary. Lach. (*r*).
— — left CHEST from ovary. Apis (*r*).
— — CRURAL region and thighs from ovary. Staph.
— — — nerve (anterior). XAN.
— — — — — (descending). Pod. (*r*).
— diagonally. Apis (*r*), Medorr. (*r*).
— downward. Medorr.
— — and forward from left ovary. Arg-m. (*l*).
— to GENITAL organs from ovary. Lach. (*r*).
— — GROIN, left. Medorr. (*l*).
— — — BUFO, Cub., Lill-t., Ustl.
— — — and hypogastrium. Xanth.
— — — or left leg. Lill-t., Ustl.
— — — thigh and leg. Ovi-gall-p. (*l*).
— through GROIN toward sacrum. Plat.
— to HIPS. Apis (*r*), Brom. (*l*), Merc., Xanth. (*r*).
— down HIPS. Ustil. (*l*).
— to *Hips* and thighs. Lill-t.
— over HIPS and down thigh. Xanth. (*r*).
— — right *hypocondrium* from. Lill-t. (*r*), (Lach. (*r*)).
— — *iliac* region and groin and sometimes into left leg. *Thuja* (*l*).
— — knee (shooting) from. Wyeth. (*l*).
— — leg (left). Apis, Cham., Lill-t., Phos., Thuja, Ustl.
— — — (right). *Apis.*
— — — (shooting down). Ustl. (*l*).
— from *left* to *right*. Apis, Lac-can., LACH., Lill-t., Syphil., [Ustl.
— to liver from. Lach. (*r*), (Lill-t. (*r*)).
— down *limbs*. Ferr-iod. (*l*), Gossip (*l*).
— to *loins*. Staph.
— — — and groins. Cub.
— from *one* to the *other*. ONOSMOD.
— outward and backward from. Sep.
— to ovaries from back of each hip. Sep.
— to ovary from left labium, through uterus. Bell., Phos., Thuja.

Extending to ovary from left leg. Lac-can. (*l*).

— — — — umbilicus. Coloc., Hydrast. (*l*).

— pubes, across from. Lill-t. (*l*).

— ribs. Apis (*r*).

— right to left. Graph., LYC., Xanth.

— to *sacrum* and thighs from. Arg-n.

— — *shoulder*-blade from. *Bor.* (*r*).

— — *shoulder* from. Pod.

— — whole left *side* from. Lac-can. (*l*), Plat. (*l*).

— toward *stomach* from. Coloc. (*l*).

— to *thighs* from region of. APIS, Arg-n., Ars., Bry., *Cact.*, *Calc-c.* (*r*), Carb-an., Cham., Lac-can., Lill-t. (*l*), Magnol. (*l*), Nat-m. (*r*), Pallad. (*r*), Phos., Pod., Staph., Xan.

Extending to *anterior* surface of THIGHS. Lill-t. (*l*)., Xanth. (*l*).

— — inner surface of *thighs*. Ars., *Lill-t.* (*l*), Phos. (*l*).

— — — — — — and down knee. Pod. (*r*).

— — outer surface of *thighs*. Lill-t.

— — *thighs* and over hip from region of. Xanth. (*r*).

— upward (shooting). Cimic., Lill-t., Lach. (*l*).

— to *uterus*, from. Ham. (*r*), Iod. (*r*), Lach. (*r*), Sep., Ustl. (*l*), Sac-lac. (*r*).

— — — through broad ligament. Iod. (*r*).

— — vagina from. Ovi-gall-p. (*l*), Sep. (*l*).

Pinching pains in region of. *Canth.*, Cham., PLAT.

Pressive pains in region of. ARS. (*r*), Lac-def., LACH. (*l*), Plat.

Pressure in. Angust. (*r*), Sep.

— above. Eupat-purp. (*l*).

— downward. *Plat.*

Pulling down sensation in. Medorr. (*l*).

— in region of. Plat.

Pulsation (including throbbing in). Calc-c., Cup., *Onosmod.*, Pod. (*r*).

— in region of. Apis, Ars., BELL. (*r*), CACT., Calc-c., COP. (*r*), Cub.

— — — at same hour every day extending to the thighs. Cact.

Pushing in region of. Caps. (*l*).

Quick pain. Eupat-purp. (*l*). (Compare Cutting.)

Rawness, sensation of, in region of. Merc. (*l*).

Right ovary. Abs., Æscul., Ang., APIS, Arg-m., *Arg-n.*, *Ars.*, BELL., Bor., Brachy., Bry., Calc-c., Carb-an., *Cenchris.*, Collins., Conval., *Coloc.*, Cop., Cub., Curar., Ferr., Fer-iod., Fluor-ac., Glon., *Graph.*, Ham., Hell., *Iod.*, Kali-bro., *Kali-iod.*, Kali-n., Lac-can., *Lach.*, Lact-ac., *Lill-t.*, Lyc., *Mag-ph.*, Medorr., Nat-m., PALLAD., Pic-ac., Plat., POD., Psor., Puls., Rhod., Rhus-t., Sac-lac., Sarsap., Sec., Sep., *Xan.*

Sensitiveness, including soreness and tenderness. Alumina, ANT-C., APIS (*r*), Arg-m., Arg-n., Artemis., Atrop. (*l*), *Bufo*, Bry. (*r*), Canth., Chin., Cimic., *Coloc.* (*l*), Con., Cup-ars. (*l*), *Graph.*, Guai., Ham., Helon., Hep., *Iod.* (*r*), Jecoris, Kali-brom. (*l*), Lac-can., LACH., LILL-T., Medorr. (*l*), NUX-M., *Onosmod.* (*l*), PALLAD. (*r*), *Plat.* (*l*), Psor. (*r*), Puls., Rhus-t. (*r*), Sec-c. (*r*), Sep., STAPH., Syph. (*l*), Tarent., *Tereb.*, Therid., Ustl. (*l*), Vesp. (*l*).

Sharp pain in. APIS (*r*), Cenchris (*l*), Kali-ph. (*l*), LILL-T., Lac-can. (*r*), LACH. (*l*), Lyc., Sep., STAPH., Ustl. (*l*), *Vibur.*

— — — region of. Hydrast. (*l*), *Lac-can.*, Lill-t., Xanth.(*r*).

Shooting in. Abrot. (*l*), Absin. (*r*), Cenchris. (*l*), Bry., LACH. (*l*), Lill-tig. (*l*), Lyc., MAG-PH. (*r*), Merc., *Pod.* (*r*), Sac-lac., Staph., Thuj. (*l*), VIBUR., Xanth. (*r*).

— across pubes. Lill-t. (*l*).

— to knee. Wyeth (*l*).

— — hip. Xan. (*r*).

— up side. Cimic.

— — left *side* and down arm. Lac-can.

Smarting in (compare Rawness). Eupat-purp. (*l*), Syph. (*l*).

Soreness. See Sensitiveness.

Sore spot, pain as of, in region of right, extending to thighs. *Bry.*

Sore, as of a. Syph. (*l*).

Spasmodic pains in. Coloc. (*l*), Mag-ph., Ustl. (*l*).

Squeezing in region of. Coloc. (*l*), THUJ. (*l*). (Compare Grasping, etc.).

Sticking in. *Sepia* (*l*).

— — region of. CAPS. (*l*), *Plat.* (*r*).

Sticking from cervix to fundus of uterus, and then to right ovary, causing nausea. Sepia.

Stinging pains in. APIS (*r*), Bry., Lill-t. (*l*), Merc., Sep.

— — in region of. Apis, Bor., Goss., Graph., LILL-T.

Stitches in. ARS. (*r*), *Bry.*, *Bufo*, *Coloc.* (*r*), Graph. (*l*), Kali-c., LACH. (*l*), Lill-t., Lyc., Merc. (*l*), Ovi-gall-p. (*l*), SEP., Staph.

— — region of. *Amb.*, ARS. (*r*), *Bell.* (*r*), Bor., Brom., Bry., Canth., *Coloc.* (*r*), Con., Graph., Ustl.

Strained pain, as if, in region of. Amm-m., Apis, *Arn.*

Suppuration. See Abscess.

Swelling (compare Enlargement). Ænanth., *Alumina*, Amm-br. (*l*), APIS (*r*), ARS. (*r*), ATROP. (*l*), Bell., BROM. (*l*), *Bufo*, CARB-AC. (*l*), Collin., COLOC. (*l*), Coni., Cub., Goss. (*l*), GRAPH. (*l*), Ham., IOD., KALI-BROM. (*l*), KALI-IOD. (*r*), LACH. (*l*), LILL-T., Nux-m., PALLAD. (*r*), Staph., Syph. (*l*), Thuj., *Ustl.* (*l*).

— sensation of. Kali-iod., *Lill-t.* (*l*).

Tearing in. Abrot. (*l*), *Graph.* (*r*), *Ham.* (Merc.).

— — region of. *Kali-iod.* (*r*), Lill-t., *Plat.* (*l*).

Tenderness. See Sensitiveness.

Tensive pain. Ant-c., ARS., *Coloc.*, LACH. (*l*), Medorr. (*r*), [PLAT.

Throbbing in. See Pulsating.

Tickling in region of. Prun-sp.

Tightness (compare tensive pain) in region of. Apis.

Tired pain in. Pod.

Tumor of. *Apis*, Apoc-can., *Ars.*, BAR-M., Calc-c., Coloc., Fluor-ac (*r*), Graph., Hep., Iod., LACH., Lyc., Merc., PLAT., POD. (*r*), Staph., Stram., Syphil., Thuj., Zinc.

Twinges of pain in region of. Pic-ac. (*l*).

Twisting in. Graph. (*r*).

Twitching in region of. Kali-iod. (*r*).

— — — — extending to back. Abrotan.

Uneasiness in region of. Lyssin (*l*).

Vitality, absence of in. Chenop-v.

Wedge-like pain from right ovary to uterus. Iod.

Weight in (compare Heaviness). APIS, Con., Kali-c., Lill-t. (*l*), Sep.

— — region of. Lac-def. (*l*).

